# Because I Got High: Marijuana Induced Pseudo-Wellen's Syndrome

**DOI:** 10.7759/cureus.10390

**Published:** 2020-09-11

**Authors:** Fadi Kandah, Sebastian Mikulic, Pujan Patel, Pooja Dhruva

**Affiliations:** 1 Medicine, University of Florida Health-Jacksonville, Jacksonville, USA; 2 Cardiology, University of Florida Health-Jacksonville, Jacksonville, USA; 3 Internal Medicine, University of Florida Health-Jacksonville, Jacksonville, USA

**Keywords:** pseudo-wellens, acute coronary syndrome, marijuana, wellens

## Abstract

Wellen’s syndrome is a pattern on ECG that signifies impending acute myocardial infarction (MI) of the proximal left anterior descending (LAD) artery. This same pattern can also be noted in several benign diseases that may mimic Wellen’s syndrome. Here we discuss a 36-year-old patient with no cardiac risk factors who presented with typical angina shortly after smoking marijuana. Upon arrival to the ED, an electrocardiogram revealed new biphasic T wave inversions in the anterolateral leads and cardiac biomarkers were found to be elevated. The patient was taken for emergent coronary angiography which revealed widely patent coronary arteries. Soon after, the patient was diagnosed with Pseudo-Wellen's syndrome secondary to cannabis use. This case report highlights the importance of identifying causes that may resemble Wellen’s syndrome, especially in young adults without risk factors for acute coronary syndrome (ACS). Recognizing these cases can help avoid further invasive diagnostic testing, along with the complications that may go along with it.

## Introduction

Wellen’s syndrome is an electrocardiographic pattern that signifies critical stenosis of the left anterior descending (LAD) artery. Two patterns may exist: Type A, which is characterized by deeply biphasic T waves in leads V2 and V3, and Type B, which is characterized by deep and symmetrically inverted T waves in the precordial leads [1]. Knowledge of this pattern alerts the attending physician of an impending acute myocardial infarction (MI) and urgent intervention is warranted. However, there are certain clinical scenarios where these patterns may be present without critical LAD stenosis, which is called Pseudo-Wellen's syndrome. Numerous etiologies have been documented in the medical literature, and some of the most common include marijuana, cocaine, Tako-Tsubo cardiomyopathy, pulmonary embolus, and congenital myocardial bridge [1]. The mechanism may be related to transient myocardial ischemia secondary to vasospasm or myocardial edema due to external insults [2].^ ^

Recognition of the Wellen’s pattern is important to diagnose an early MI and can be argued that the pattern is high risk and should be an ST-segment elevation myocardial infarction (STEMI) equivalent. One study showed that out of 145 patients admitted with unstable angina, 26 showed a Wellen’s pattern on EKG and 75% who were not operated on developed extensive anterior wall infarction within a few weeks after admission [3]. This data is supported in many other studies which understandably leads to a large number of urgent interventions for this EKG sign. However, it is vital to interpret these EKG signs in the correct clinical context. In patients without cardiac risk factors, it is reasonable to conduct a thorough investigation for causes that may mimic Wellen's to avoid the burden of further invasive testing and correctly treating the underlying cause. Here, we describe a young gentleman who presented with Pseudo-Wellen's syndrome following heavy marijuana use. 

## Case presentation

**Figure 1:**
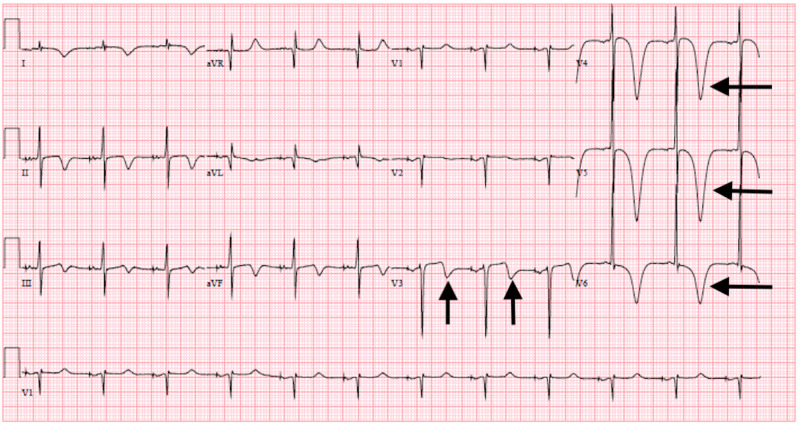
EKG demonstrating Wellen’s type pattern: biphasic T waves seen in lead V3 and deeply inverted T waves seen in leads V4-V6

A 36-year-old male with sick sinus syndrome presented with typical angina that began while he was smoking marijuana. On arrival, the patient’s vitals were within normal limits. He described the chest pain as substernal, pressure-like which radiated across both sides of the chest wall. There was associated nausea and diaphoresis involved. The pain lasted approximately one hour and was not relieved by nitroglycerin. The patient denied having similar prior chest pain. He reported not taking any medications at home. His family history was unremarkable for cardiac disease. He stated that he smoked a few cigarettes a week and did not drink alcohol. He admitted to using marijuana daily but denied any other illicit drug use. A drugs of abuse screen (DOA) was performed which was cannabinoid positive. Physical exam revealed an alert and oriented male. His cardiac examination was significant for mild tachycardia of 102 beats per minute with regular rhythm, distinct S1/S2 with no murmurs, gallops or rubs. Lungs were clear to auscultation bilaterally. Extremities showed no peripheral edema. The initial EKG showed diffuse biphasic T wave inversions in leads V3-V5 as well as elevation of cardiac biomarkers (initial troponin T of 0.15 ng/mL), consistent with Wellen’s syndrome (Figure 1).

**Figure 2:**
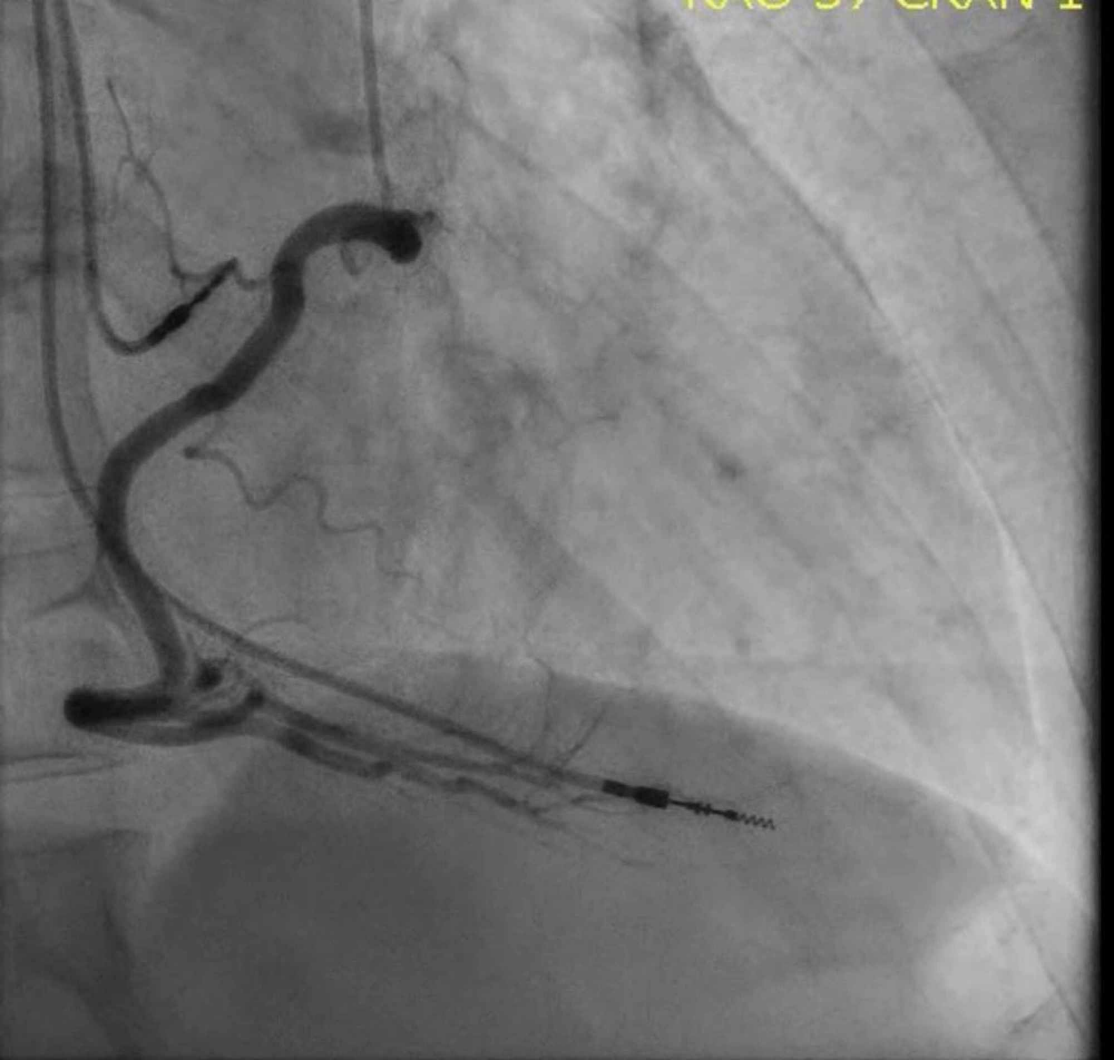
Left heart catheterization (right coronary artery) showing angiographically normal coronary vessels

**Figure 3:**
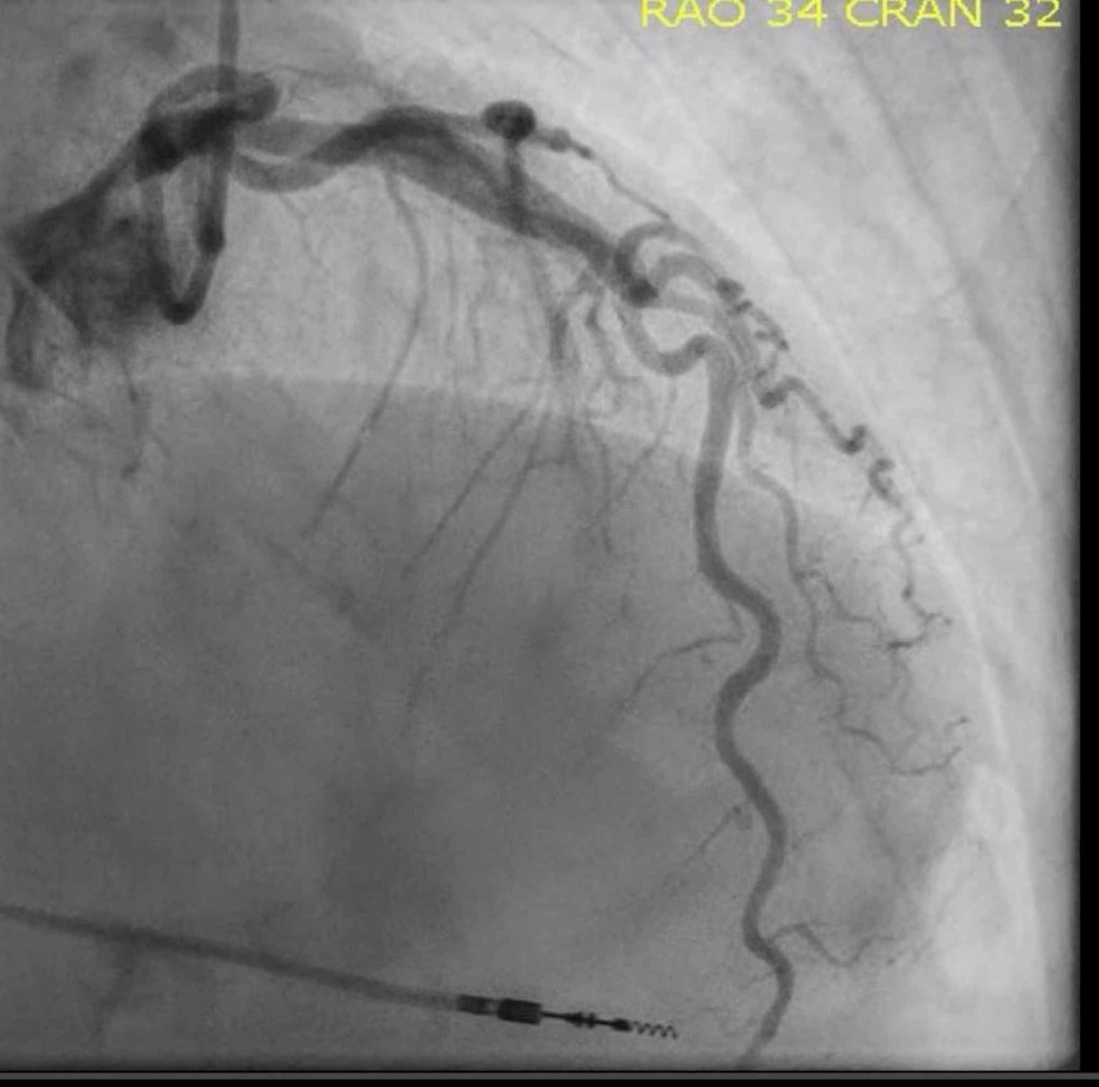
Left heart catheterization (left anterior descending and left circumflex coronary artery) showing angiographically normal coronary vessels

He was given 325 mg aspirin as well as sublingual nitroglycerin with mild relief in symptoms. Due to his typical chest pain, EKG changed, and he had elevated troponin level; it was decided to send the patient for an urgent left heart catheterization. The catheterization revealed widely patent coronary arteries without evidence of vasospasm (Figures 2-3).

The patient stated that his chest pain resolved after returning from the catheter lab. Repeat EKG was done showing consistent T wave inversions. Two more sets of cardiac biomarkers were downtrending. Transthoracic echocardiography revealed an ejection fraction of 65% with no structural or valvular abnormalities. A cardiac MRI subsequently did not reveal enhancement and ruled out myocarditis or infiltrative disease. Serial EKGs showed resolution of the T wave inversions and the patient did not have any more episodes of chest pain.

## Discussion

This case demonstrates Pseudo-Wellen’s syndrome induced by marijuana use. Review of literature shows a paucity of cases describing Pseudo-Wellen's syndrome secondary to marijuana ingestion. Wellen's syndrome is defined as angina with EKG showing biphasic or deeply inverted T waves in V2-V4 [3]. The EKG pattern is part of the ominous syndrome associated with critical occlusion of the proximal left anterior descending artery. 

The initial presentation of Wellen's syndrome will be of suspected acute coronary syndrome (ACS) based on a structured diagnostic approach including history, physical examination, EKG, and cardiac biomarkers. While obtaining patient history, traditional risk factors (e.g. sex, diabetes, hypertension, age, drug use, family history) should be evaluated for appropriate risk stratification. The description of the angina can also help narrow the differential, along with identifying associating symptoms such as nausea, vomiting, diaphoresis, and shortness of breath. The EKG is paramount to the diagnosis and will reveal the classic findings of Wellen's syndrome; symmetric and deeply inverted T waves in the precordial leads V1-V4, or biphasic T waves in leads V2-V3. Of note, there should be no loss of R wave progression and no pathologic Q waves [4]. These findings alone will alert the experienced physician to suspected critical LAD stenosis and impending MI with evaluation for urgent cardiac catheterization. In Pseudo-Wellen’s syndrome, a diagnosis of exclusion, coronary angiography will reveal patent coronary arteries [5].^ ^

Differential diagnoses in a young adult with similar presentation (aside from ACS) should include myocarditis or infiltrative disease, such as sarcoidosis or amyloidosis [6].According to one study, cardiac amyloidosis can present as an ACS like picture with elevated troponins and angina, as well as nonspecific EKG changes, suggesting pseudo-infarction. Cardiac sarcoidosis usually presents between the ages of 25-45 years of age. However, in these patients, EKG findings may reveal a conduction abnormality. Cardiac MRI with global transmural or subendocardial late gadolinium enhancement is a classic finding of both infiltrative diseases [7]. In Wellen’s syndrome, EKG changes with deep T wave inversions can persist for several days. Deep T wave inversions can last hours to weeks, even during chest pain free periods. These changes can last until definitive management of the stenosis is done. In Pseudo-Wellen’s, it is more difficult to give a definite timeline on when the EKG changes will resolve, especially when it is attributed to substance abuse. In one case report, a 41-year-old patient with chest pain presented with deep T wave inversions in leads V1-V4. He was positive for cocaine on his urine drug test and cardiac biomarkers were negative. The patient would undergo an urgent coronary angiography which also presented with normal coronary anatomy [8].

To date, there are no guidelines on the management of a Pseudo-Wellen’s syndrome as the only way to confirm the diagnosis is with a left heart catheterization. This presents the clinician with a unique situation: pursue invasive measures or manage the patient conservatively. 

While data is lacking in this scenario, it can be argued to avoid angiography in a young patient without cardiac risk factors, and simply to follow with serial EKGs. As demonstrated with our patient (as well as other case reports), resolution of the ST segment changes will occur after a short period of time [5,8]. This is also not solely seen in cases of substance abuse. Other considerations should be taken into account, such as metabolic, hematologic, or infectious causes. An example is seen in a case report of a 45-year-old male who presented with epigastric pain and pressure like left-sided chest pain. The patient had normal troponin levels but EKGs showed findings of dynamic T wave changes consistent with Wellen's syndrome. He was taken for emergent left heart catheterization which demonstrated normal coronary arteries. He was found to have significantly elevated lipase and his symptoms resolved with treatment for acute pancreatitis. His EKG findings also resolved within twenty-four hours [9]. Other causes of Pseudo-Wellen's syndrome that have been shown in the literature include left ventricular hypertrophy, sepsis, pulmonary embolism, and significant hypertension [5,7,9]. 

We propose that management of suspected Pseudo-Wellen's syndrome in a young adult should focus on supportive care, administering nitroglycerin as needed for chest pain, avoiding beta blockers in cocaine-induced causes (to avoid the unopposed alpha adrenergic effects), examination for other etiologies that may mimic Wellen’s syndrome on ECG, and pursuing further invasive measures based on risk stratification. Following this strategy not only avoids unnecessary diagnostic testing, but it also limits financial cost and psychological burden to the patient.

## Conclusions

With the increasing use of marijuana in the general population, general practitioners and emergency medicine providers should be cognizant of ACS mimicking clinical scenarios that may lead to further invasive workup. It is important to note that since 2012, marijuana use has increased in young adults. Ten states have legalized recreational marijuana (as well as the District of Columbia), and this number is expected to increase in the near future. Therefore, the number of cases of Pseudo-Wellen’s syndrome due to marijuana use can also be expected to increase. Further investigation is required for the socioeconomic and health implication of this rare syndrome. 
